# CD4+CD25+CD127^low^ Regulatory T Cells Play Predominant Anti-Tumor Suppressive Role in Hepatitis B Virus-Associated Hepatocellular Carcinoma

**DOI:** 10.3389/fimmu.2015.00049

**Published:** 2015-02-25

**Authors:** Shreya Sharma, Ritu Khosla, Paul David, Archana Rastogi, Ashish Vyas, Dileep Singh, Ankit Bhardwaj, Amrish Sahney, Rakhi Maiwall, Shiv Kumar Sarin, Nirupma Trehanpati

**Affiliations:** ^1^Department of Research, Institute of Liver and Biliary Sciences, New Delhi, India; ^2^Department of Pathology, Institute of Liver and Biliary Sciences, New Delhi, India; ^3^Department of Hepatology, Institute of Liver and Biliary Sciences, New Delhi, India

**Keywords:** T regulatory cells, hepatitis B virus, hepatocellular carcinoma, transforming growth factor-β, alpha-fetoprotein

## Abstract

**Background:** Hepatocellular carcinoma (HCC) is the second leading cause of cancer death worldwide and hepatitis B is one of the commonest causes. T regulatory cells (Tregs) are strong immunomodulators and are likely to play a major role in HCC development. HBV infection is reported to induce expansion of Tregs. We investigated the CD4+CD25+CD127^−ve^FoxP3+ Tregs in HBV-related HCC as compared to non-HBV-HCC.

**Patients and Methods:** Whole blood immunophenotyping was analyzed by multicolor flow cytometry in patients with HBV-related HCC (HBV-HCC, *n* = 17), non-HBV-HCC (*n* = 22; NASH = 16, alcohol-related = 6), and chronic hepatitis B infection (CHBV; *n* = 10). Tregs functionality was checked by *in vitro* suppression assays using CD4+ CD25+ CD127^low^ Tregs. Levels of serum alpha-fetoprotein (AFP), expression of FoxP3, IL-10, PD1, TGF-β, and Notch in Tregs, and liver explants were analyzed by flow cytometry, immunohistochemistry, and quantitative RT-PCR.

**Results:** CD4+CD25+^hi^ and Foxp3 expression in CD4+CD25+^hi^CD127^low^ was significantly increased (*P* = 0.04, *P* = 0.007) in HBVHCC compared to non-HBVHCC and CHBV patients. HBVHCC also showed high IL-10 and TGF-β secreting CD4 + CD25 + ^hi^Tregs. The PD1 expression in CD4 + CD25+^hi^ was significantly decreased in the HBVHCC than non-HBVHCC. In HBVHCC, AFP levels were significantly high (median 941, range 2–727940) than non-HBVHCC (median 13.5, range 2–18,900). In HBVHCC, patients with high AFP (range; 3982–727940 ng/ml) showed positive correlation with Foxp3 expression in CD4+CD25+^hi^ CD127^low^ (*r* = 0.857, *P* = 0.014). Reduced PD1 expression in HBVHCC also had negative correlation with FOXP3 in CD4+CD25+^hi^ CD127^low^ (*r* = −0.78, *P* = 0.04). However, AFP levels in non-HBVHCC showed negative correlation with (*R* = −0.67, *P* = 0.005) with CD4+CD25+^hi^ Tregs.

**Conclusion:** Our results demonstrate that CD4+ CD25+^hi^ Tregs from HBVHCC patients have decreased expression of PD1, resulting in higher IL-10 and TGF-β secretion. Increased suppressive ability of Tregs in HBV-related HCC confers increased anti-tumor suppressive response than in non-HBV-HCC. Modulation of Tregs and PD1 may serve as useful therapeutic targets.

## Introduction

Hepatocellular carcinoma (HCC) is the second most common cancer worldwide and its incidence in Asia is on a rise (1). The most important causes leading to HCC are HBV and HCV infections, autoimmune hepatitis, heavy alcohol consumption, aflatoxin B1, obesity, iron overload, age, and gender (males are more susceptible than females). However, hepatitis B virus-related chronic liver disease is the most important risk factor for development of HCC. Importantly 50–60% of HCC in Asia is associated with chronic HBV infection (2–4).

Clearance of hepatitis B infection is T cell dependent and during acute infection, T cell responses are vigorous, polyclonal, and multi-specific. However, in chronically infected patients, T cell responses are relatively weak and narrowly focused. Apparently, CD8+ T cells are the key cellular effectors mediating HBV clearance from the liver and CD4+ T cells help them to clear the virus. Regulatory T cells (Tregs) play an important role in regulating the immune system by suppressing self-reactive CD4 and CD8 T cells (5–7).

Naturally occurring and inducible Tregs exert their suppressive effects either via cell to cell contact by membrane-bound molecules or through contact-independent manner mainly by release of IL-10 and TGF-β cytokines (8). TGF-β and IL-10 are responsible for the suppression of anti-tumor immune responses and therefore lead to successful tumor escape (9). During chronic inflammation, induction of Tregs happens through activation of Notch and Wnt signaling (10–12).

T regulatory cells (Tregs) in periphery as well as in tumor area express more of Foxp3 and specifically inhibit CD8 T cell activity, thereby blocking virus-specific immune responses and leading to viral persistence (13–18). During chronic hepatitis B infection, frequency of circulating Tregs increases, which are able to modulate virus and tumor antigen-specific immune responses (19, 20). However, increase of Tregs is inversely proportional to HBV DNA titers. In the later stages of infection, during fibrosis and cirrhosis, abundant TGF-β favors the differentiation and expansion of Tregs. In cirrhotic patients, Treg frequency increases in both peripheral blood and liver compared to non-cirrhotic patients. Therefore, Tregs-mediated immunosuppression contributes to ideal microenvironment for oncogenic transformations (21). In addition, Tregs with increased FOXP3 and CTLA4 expression in tumor microenvironment show marked elevation in the ratio of TGF-β/IL-17 (22).

However, the precise mechanism of regulatory T cells in HBV-induced HCC has not been compared with non-HBV-related HCC. Therefore, we undertook to compare the CD4+CD25+CD127^low^ regulatory T cells and their regulation by TGF-B, IL-10, and PD1 in patients with HBV induced HCC vs. non-HBV-related HCC.

## Patients and Methods

### Patients

Hepatocellular carcinoma (HCC) patients were diagnosed based on classical radiological features of arterial enhancement and venous washout with raised alfa-feto protein, and if needed, histological confirmation on biopsy or surgical specimens was done.

Hepatocellular carcinoma patients were divided into two categories: hepatitis B virus-infected (HBV-HCC; *n* = 17) and non-HBV-HCC (*n* = 22; cryptogenic = 16, NASH = 4, ALD = 2).

Treatment naïve chronic hepatitis B patients (CHB, *n* = 10) HBsAg with raised ALT, HBsAg+ for more than 6 months and with histological evidence of chronic hepatitis.

#### Exclusion criteria

The patients with daily alcohol consumption in last 1 month, diabetes, severe systemic illness, pregnancy, co-infection with HIV, or other hepatic viruses, or receiving immunosuppressive therapy for other associated illness were excluded. The study was approved by the Institutional Ethics Committee and informed consent was obtained from each patient.

Whole blood was collected in k3 EDTA vials. Tissues from HBV-HCC and non-HBV-HCC patients, undergoing liver transplant/resection were collected and stored in liquid nitrogen. Tissue piece to be used for RNA isolation was stored in RNA later at −20°C.

### Flow-cytometric analysis

Whole blood was permeabilized and fixed using cytofix/cytoperm (BD Pharmingen, San Jose, CA, USA) according to manufacturer’s protocol followed by staining for 20 min at room temperature in the dark with cocktail of antibodies including anti-CD4-APC, anti-CD25-FITC, anti-FOXP3-PE, anti-PD1-PeCy7, anti-IL10-APC, anti-CD127-APC, anti-CD8PeCy5, anti-NOTCH1 PE, and anti TGF-β APC (BD Pharmingen, CA, USA). After staining, RBCs were lysed using BD FACS lysing solution (BD Pharmingen, San Jose, CA, USA) as per manufacturer’s instructions.

Anti-Notch1 PE antibody (clone mN1A) was procured from eBiosciences, CA, USA; mN1A antibody reacts with the intracellular domain of human Notch1. The mN1A antibody has a low affinity for the full-length (unprocessed or heterodimeric cell surface) forms of Notch1. Therefore, Notch1 expression was considered intracellular not surface expression.

More than 50,000 cells were acquired for flow-cytometric analyses on BD FACS Caliber and the results were analyzed using the TreeStar Flow-Jo software version 8.8.7.

### Isolation of PBMCs

Ten to fifteen milliliters of blood samples were centrifuged at 800 g for plasma separation. After removing plasma, pooled blood was diluted in cold PBS and PBMCs were isolated by Ficoll–Hypaque density gradient centrifugation. PBMCs were suspended in RPMI1640 medium supplemented 10% fetal bovine serum (FBS) for further use. The viability of isolated cells was determined by trypan blue exclusion staining.

#### CD4+CD25+CD127^low^ T regulatory cell isolations

Ten to fifteen milliliters of whole blood was processed for isolation of CD4+CD25+CD127^low^ regulatory T cells using manufacturer’s protocol (Stem cell technology, Vancouver, BC, Canada).

### *In vitro* suppression assays using CFSE

Freshly isolated CD4+CD25+CD127^low^ Tregs were used directly to assess their functional suppressive capacity. CD4+CD25− cells were stained with 500 nM CFSE and incubated for 12 min at 37°C. After incubation, cells were washed twice with pre-warmed 1640 RPMI culture medium. After counting, the cells are ready for use. CD4+CD25+CD127^low^ Treg cells from HBV-HCC and non-HBV-HCC patients were co-cultured with CFSE labeled CD4+CD25− cells from the same patient at ratios 1:10. CFSE labeled CD4+CD25-CD127^low^ cells without Treg cells were used as control. Cells were stimulated by anti-CD3 (1 μg/ml) and anti-CD28 (1 μg/ml) and cultured in RPMI 1640 medium (HiMedia) supplemented with 10% FBS and 1 penstrep for 72 h at 37°C with 5% CO_2_. Proliferation of CD4+CD25−CD127^low^ cells was determined by measuring CFSE dilution with flow-cytometric analyses using BD FACS Calibur and the results were analyzed using the TreeStar Flow-Jo software version 8.8.7.

#### Total RNA isolation

Extraction of total RNA was done from PBMCs, CD4+CD25^high^ CD127^low^ Treg cells, CD4+CD25− cells, and resected liver tissues using MIRVana kit (Ambion, Austin, TX, USA). The concentration of RNA was measured using Nanodrop ND-1000 (ThermoScientific, USA). A total of 1–2 μg of the RNA was used for cDNA preparation using random hexamer primers.

### Quantitative real-time PCR

qRT-PCR was performed for CD25, TGF-β, IL-10, PDL1, PDL2, FOxP3, Notch, and wnt signaling molecules using SYBR Green PCR Kit (Applied Biosystems, USA) and ABI PRISM 7700 Sequence Detector with ViiA 7 software (Applied Biosystems, USA). The primers of all genes were designed using Primer 3 software (Table 1). The gene expression level was normalized against 18S (control gene) RNA. Relative gene expression values expressed as fold change were subsequently determined using the 2^−ΔΔCT^ method.

**Table 1:** **Oligo sequences used in the study for quantitative RT-PCR**.

S. no.	Gene	Oligo sequence
1	IL-10	5′-CCGCCTCAGCCTCCCAAAGT-3′
		5′-CCCTAACCTCATTCCCCAACCAC-3′
2	TGF-β	5′-GAGGCGCCCGGGTTATGCTGGTTG-3′
		5′-CGCAAGGACCTCGGCTGGAAGTGG-3′
3	CD25	5′-TGGACACACAAGGTGCAA-3′
		5′-TGTGACCTCCATCCCTTCTC-3′
4	FoxP3	5′-CACCTGGCTGGGAAAATGG-3′
		5′-GGAGCCCTTGTCGGATGAT-3′
5	Wnt 3a	5′-CGCGAGTCGGCCTTCGTTCA-3′
		5′-AGGCGGCCCCTTATGATGCG-3′
6	Cyclin D1	5′-CTCCATCCAGGGATTCTTCA-3′
		5′-TTTTTGGAGCTTCTGGCTGT-3′
7	β-catenin	5′-GACAGCAATCAGCTGGCCTGGT-3′
		5′-ACCACTCCCACCCTACCAACCA-3′
8	Notch 1	5′-CGGGTCCACCAGTTTGAATG-3′
		5′-GTTGTATTGGTTCGGCACCAT-3′
9	Notch 2	5′-GTGCAGGAATTGGAAAGTTGGA-3′
		5′-GGCCGCTTCAGAGGAAAAG-3′
10	Notch 3	5′-GCCATCTCCCTTTGGGAACT-3′
		5′-CCACATTTACAGGGACATAAAGGA-3′
11	Notch 4	5′-CCAAGAAATGCCCATAAACCAA-3′
		5′-GCCTTTTAATGGGTAATCATTTTTG-3′
12	Jagged 1	5′-CCAGGTCTTACTACGGAGCACATT-3′
		5′-CGCAAGCGATGTAGATTGAATATT-3′
13	Hey 2	5′-TACTTTGACGCACACGCTCT-3′
		5′-CGCAAGTGCTGAGATGAGAC-3′
14	Hes1	5′-GGACATTCTGGAAATGACAGTGAA-3′
		5′AGCGCAGCCGTCATCTG-3′

### Immunohistochemical analysis

Immunohistochemistry staining was performed on 3 μm sections of paraffin-embedded resected liver tissue specimen for PD1, CD25, FOXP3, PDL1, and TGF-β in HBV-HCC (*n * = 5) and non-HBV-HCC (*n * = 5) patients.

Sections were stained with chromogen DAB (DAKO, Suyog Diagnostics Pvt. Ltd., Mumbai, India) and counterstained with hematoxylin. The condition for use of primary polyclonal antibodies were optimized and PD1 (Santa Cruz Biotechnology) FoxP3, PDL1, and TGF-β (Abcam, St Louis, MO, USA), were used at the 1:25 and 1:50 dilution, while CD25 antibody was ready to use antibody. Cellular localization, cytoplasmic, and nuclear positivity of the respective protein expression was carefully observed.

### Statistical methods

All the data comparisons are expressed as mean ± SD or median with range. The continuous data were compared using one way ANOVA or Kruskal–Wallis Test followed by *post hoc* comparison by Bonferroni method. Spearman’s correlation was used to calculate correlation in between parameters and also with alpha-fetoprotein (AFP) values. The significance is indicated with a *P* value <0.05.

## Results

### Clinical and virological characteristics of subjects

**Figure 1:**
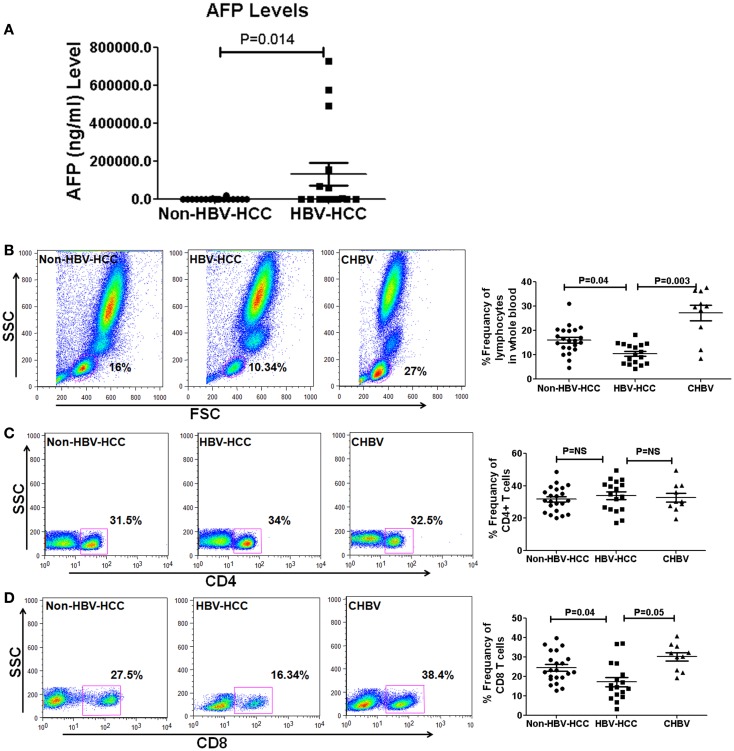
**(A)** Alpha-fetoprotein level is a predictive marker of HCC and its values are high in HBVHCC than non-HBV-HCC. **(B–D)** Representative flow-cytometric dot plots and scatter plots showing differential frequencies of total lymphocytes, CD4, and CD8 T cells. Total lymphocyte counts and CD8+ T cells were decreased in HBV-HCC than non-HBV-HCC and CHBV. There was no significant difference in CD4+ T cell counts in between all the three groups.

The clinical and virological characteristics of 49 patients are shown in Table 2. There were no significant differences in the age and sex in all groups. However, AFP levels were high in HBV-HCC (mean, 123077 ± 234626 vs. 1339 ± 4448 (Figure 1A) and median 941, range, 2–7,279,40 ng/ml in HBV-HCC vs. median 13.5 range 2–18,900 ng/ml, *P* = 0.03 in non-HBV-HCC patients (Table 2).

**Table 2:** **Clinical and virological characteristics of subjects recruited in the study**.

Parameters	Non-HBV-HCC	HBV-HCC	CHBV	*P* value
				*Non-HBV-HCC vs. HBV-HCC **HBV-HCC vs. CHBV
Age (years), mean ± SD	59 ± 8.5	54 ± 10	36 ± 13.6	NS
Sex (M:F)	17 : 5	14 : 3	9 : 1	NS
AST (IU/ml), median (range)	97.5 (71–763)	109.5 (73–229)	70.5 (41–966)	NS
ALT (IU/ml), median (range)	73 (51–402)	71 (51–229)	72 (46–872)	NS
HBsAg	Non-reactive	Reactive	Reactive	–
HBeAg	–	–	Reactive	–
AFP (ng/ml), median (range)	13.5 (2.4–18,900)	943.5 (2.24–575736.6)	38.2 (2.45–120.6)	0.047*, 0.045**
HBV DNA (IU/ml), median (range)	–	1645 (77.6–7.52 × 10^6^)	20.6 × 10^6^ (10–1.10 × 10^8^)	0.04**

*P value: Non-HBV-HCC vs. HBV-HCC; **P value: HBV-HCC vs. CHBV.

### Decreased total lymphocytes and CD8+ T cells in HBV-HCC than non-HBV-HCC patients

Flow-cytometric analysis in whole blood revealed that total lymphocytes were significantly lower in HBV-HCC compared to both CHBV (*P* = 0.003 and *P* = 0.04, Figure 1B) and non-HBVHCC patients. There was no significant difference in the frequencies of CD4+ T cells in all three groups (Figure 1C). CD8+ T cells were also significantly lower in HBV-HCC compared to both CHBV and non-HBVHCC patients (*P* = 0.003 and *P* = 0.04, Figure 1D).

### Higher regulatory T cells in HBV-HCC patients

**Figure 2:**
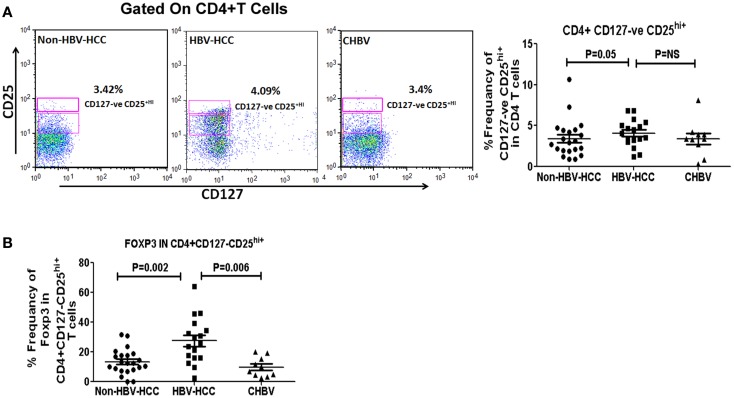
**(A)** CD4+CD25+CD127^low^ Tregs were analyzed using CD25 and CD127 markers on CD4+ gated population of T cells. Representative flow-cytometric dot plots and scatter plot showing the frequencies of CD4+CD25+CD127^low^ Tregs in all three groups. **(B)** Scatter plot shows increased expression of FoXP3 positive CD4+CD25+CD127^low^ Tregs in HBV-HCC than non-HBVHCC (*P* = 0.002).

Intra-cytoplasmic expression of FoxP3 is key regulatory factor for Tregs, therefore, we have assessed Foxp3 expression in CD4+ CD127^low^ CD25+^hi^ in HBV-HCC and non-HBV-HCC patients. Frequencies of CD4+ CD127^low^ CD25+^hi^ cells were significantly high (*P* = 0.05) in HBV-HCC patients than non-HBVHCC patients. Further, key regulatory expression of Foxp3 in CD4+CD127^low^CD25+^hi^ was also significantly high in HBV-HCC compared to non-HBV-HCC (*P* = 0.002; Figures 2A,B).

Foxp3 expression in CD4+ CD127^low^ CD25+^hi^ was significantly correlated with both low (<1000, *r* = 0.857, *P* = 0.014) and high (>1000, *r* = 0.914, *P* = 0.000) AFP values in HBV-HCC patients, which was not correlated in non-HBV-HCC patients (Tables S1–S3 in Supplementary Material).

There was no difference in frequencies of CD8+CD25+ ^hi^ in HBV-HCC than non-HBVHCC patients (*P* = NS, Figures S1A,B in Supplementary Material) but in comparison to CHBV patients, CD8+ Tregs frequencies were slightly higher in HBV-HCC patients (*P* = 0.05). However, Foxp3 expression in CD8+CD25+^hi^ was significantly high in HBV-HCC than non-HBV-HCC (*P* = 0.04) and CHBV patients (*P* = 0.002, Figure S1B in Supplementary Material).

### CD4+CD25+CD127^low^ Tregs in HBV-HCC patients are functionally more suppressive than non-HBV-HCC patients

**Figure 3:**
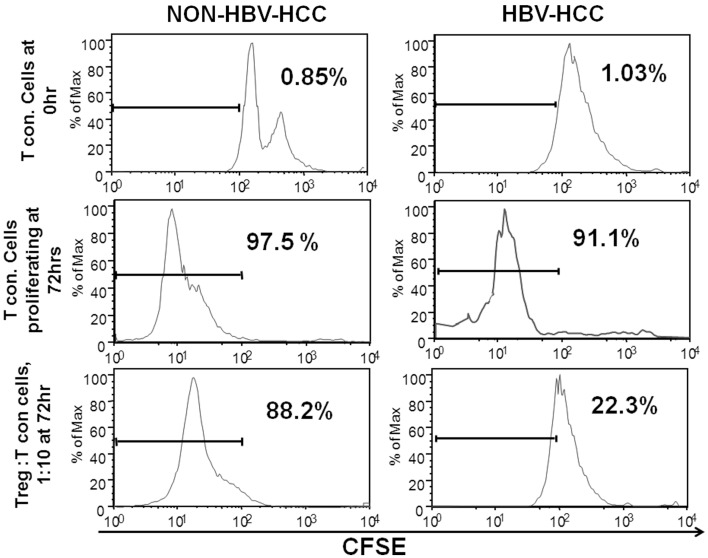
**Tregs from HBV-HCC are functionally more suppressive**. T conventional cells from HBV-HCC and non-HBV-HCC patients were CFSE labeled and cultured for 72 h with patient matched Tregs (1:10 for Tregs: Tcon) to assess CFSE dilution. Tregs from HBV-HCC showed significantly high suppression of Tcon after 72 h of co-culture (right panel) as compared to Tregs from non-HBV-HCC.

In order to assess the ability of regulatory T cells to suppress other antigen-specific or non-specific T cells, *in vitro* suppression assay was set up to analyze the suppressive ability of CD4^+^CD25^+^CD127^lo^ cells (Tregs). In this assay, T cells from HBV-HCC and non-HBVHCC patients were CFSE labeled (Tcon) and co-cultured with and without Tregs at a concentration of 1:10 (Tregs: Tcon) for 72 h to assess CFSE dilution. In both groups, Tcon alone were proliferated enough in 72 h indicated by CFSE dilution of 97.5 and 91.1%. However, after 72 h of co-culture with Tregs from HBV-HCC and non-HBV-HCC patients, Tcon proliferated less and there was only 22.3% dilution of CFSE as compared to 88.2% in non-HBVHCC patients. This suggests that Tregs from HBV-HCC inhibited the proliferation of Tcon more than non-HBVHCC patients (Figure 3).

**Figure 4:**
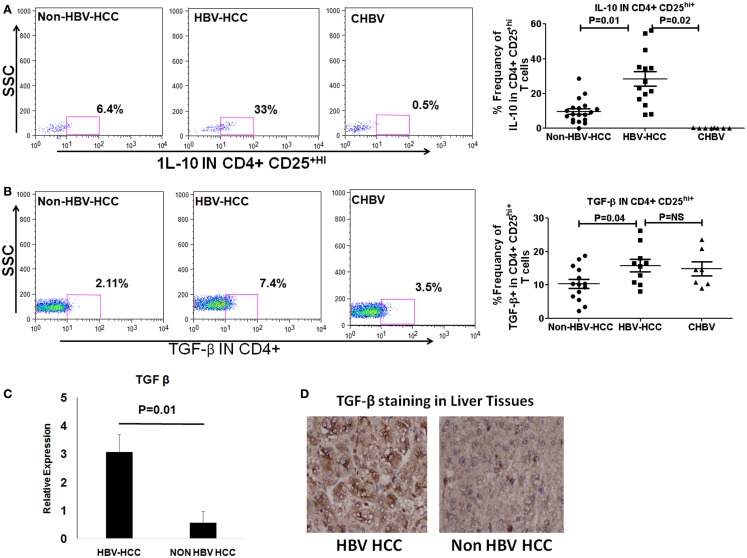
**(A,B)** Representative flow-cytometric dot plots showing the expression of IL-10 and TGF-β in Tregs in three patient groups. Scatter plot shows that both IL-10 and TGF-β were expressed significantly more in Tregs of HBV-HCC patients than both non-HBV-HCC and CHBV. **(C)** qRT-PCR analysis confirmed the overexpression of TGF-β in HBV-HCC than non-HBV-HCC. **(D)** Immunohistochemistry analysis also confirmed the over expression of TGF-β in HBV-HCC than non-HBV-HCC liver tissues. 40× magnification was used.

T regulatory cells exert their immunosuppressive activity through production of cytokines like IL-10 and TGF-β or through Notch-mediated cell to cell contact. We observed by flow cytometry, that intracellular secretion of IL-10 and TGF-β was higher in HBV-HCC Tregs than non-HBVHCC (IL-10; *P* = 0.01; and TGF-β; *P* = 0.04, Figures 4B,C). QRT-PCR and immune histochemical analysis showed the increased expression of TGF-β in HBV-HCC Tregs than non-HBVHCC Tregs (*P* = 0.01, Figures 4C,D). Secretion of IL-10 was also increased in HBV-HCC Tregs compared to CHBV patients also (IL-10; *P* = 0.02; Figure 4A).

Expansion of Tregs is driven by activation of Notch signaling also. Therefore, we have assessed Notch expression by qRT-PCR as well as by intracellular staining of Notch1 in HBV-HCC Tregs. Flow-cytometric analysis revealed that intracellular expression of Notch1 was significantly increased in HBV-HCC than non-HBVHCC Tregs (*P* = 0.048; Figure S2 in Supplementary Material).

**Figure 5:**
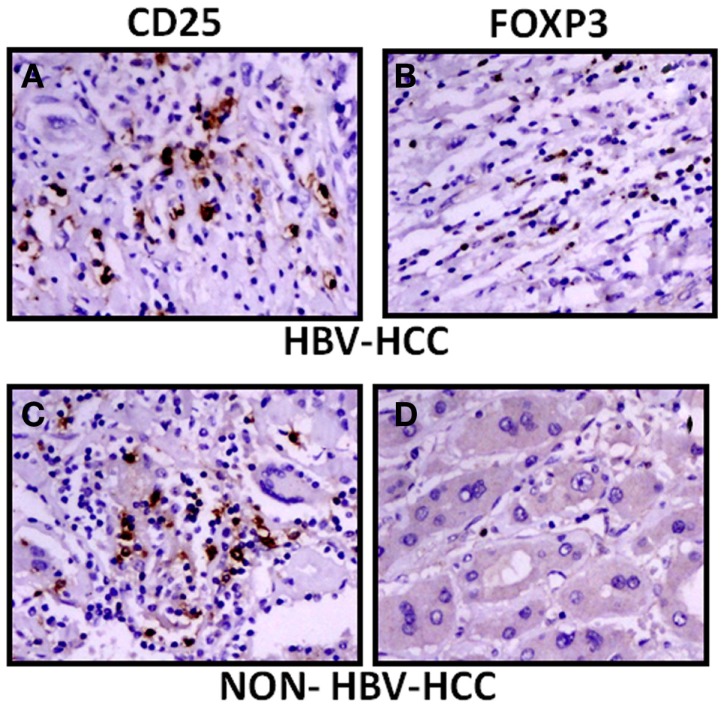
**Immunohistochemical analysis showed increased expression of CD25 and FoxP3 in HBV-HCC patients (A,B) than non-HBV-HCC patients (C,D), Magnification: 40×**.

The expression of CD25 and FoxP3 was further analyzed by immunohistochemistry in paraffin-embedded sections of HBV-HCC and non-HBVHCC cases. Both CD25 and FoxP3 were expressed more in HBV-HCC than non-HBV-HCC (Figure 5).

### Decreased programed cell death molecule PD1 in HBV-HCC Tregs

**Figure 6:**
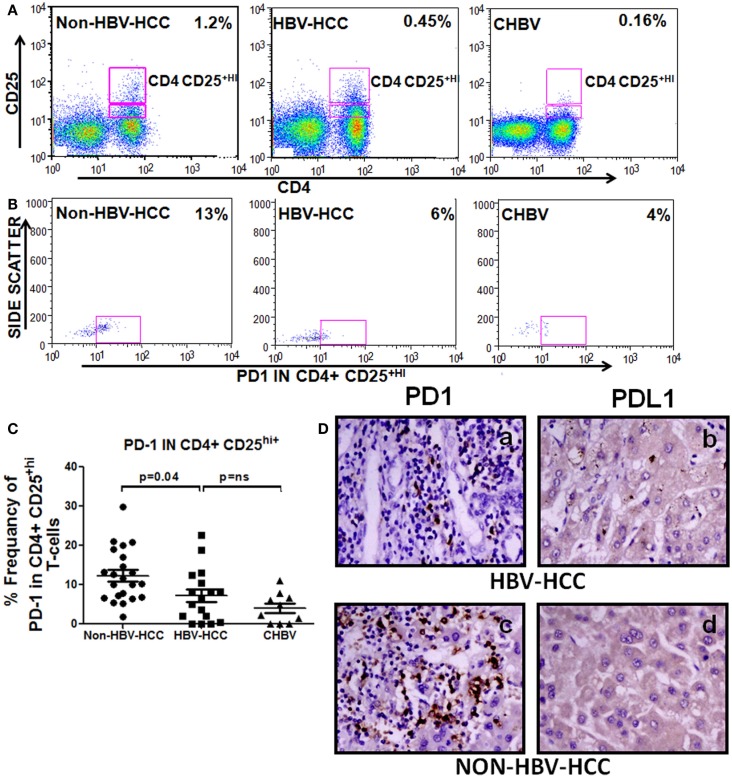
**(A–C)** Representative flow-cytometric dot plots and scatter plot showing increased expression of PD1 on CD4+CD25+ Tregs of non-HBV-HCC patients than HBV-HCC patients. **(D)** Immunohistochemistry on paraffin-embedded sections confirming the increased expression of PD1 and PDL1 (c,d) in non-HBV-HCC than HBV-HCC (a,b) patients. Magnification: 40×.

To check the differentiation status of Tregs and also as inhibiting marker, we have analyzed the expression of PD1 in CD4+CD25+^hi^ Tregs, PD1 showed lower expression in Tregs of HBV-HCC than non-HBV-HCC compared to HBV-HCC patients (Figure 6).

PD1 expression is negatively correlated in patients with low AFP values (*r* = −0.778, *P* = 0.039) in HBVHCC patients; however, no such correlation was made in non-HBV-HCC patients (Table S1 in Supplementary Material).

Immunohistochemistry also showed a decreased expression of PD1 in HBV-HCC (+) patients than non-HBVHCC patients (+ + +) (Figure 6); however, there was no significant difference in the expression of PDL1 (ligand for PD1) in HBV-HCC than non-HBVHCC patients.

## Discussion

Chronic HBV infection plus long-term recurrent immune-mediated liver damage contributes to the development of HCC. Tregs-mediated suppression potentially hinders an effective immune response, which is crucial for elimination of tumors and infection (23). However, how Treg impairs the host immune to favor tumor growth is not clearly understood in various etiology-based HCC.

In this study, we observed that HBV-HCC patients have an increased percentage of FoxP3+CD4+CD25+CD127^low^ T cells phenotypes. AFP, which is a HCC marker often associated with tumor size, was also significantly high in HBV-HCC patients than non-HBVHCC patients. Furthermore, high AFP levels in HBV-HCC patients showed significantly positive correlation with Foxp3 expression in CD4+CD25+^hi^CD127^low^ cells (*r* = 0.857, *P* = 0.014), while in non-HBVHCC cases no such correlation could be made. In other study also, proportion of CD4+CD25^high^FOXP3+ Tregs was significantly higher in patients with high serum AFP levels (24). However, AFP correlation with Tregs is controversial. In one of the study, poor correlation between CD4+CD25+ Tregs and tumor marker AFP was reported (25).

In addition, our study showed inverse relation with high Tregs and decreased CD8+ effector T cells in HBV-HCC than non-HBV-HCC patients. Further, CFSE assay showed that Tregs from HBV-HCC patients could significantly reduce the proliferation of effector T cells in comparison to those isolated from non-HBV-HCC patients. In fact, earlier studies have also proposed that both circulatory and intra-tumoral Tregs may promote HCC progression by decreasing and impairing the effector functions of CD8+ T cells (23, 26).

TGF-β and IL-10 are major cytokines through which the Tregs exert their suppressive function (27). Importantly, TGF-β produced by iTregs can induce other naive CD4+CD25− cells to become similar suppressor cells (28) whereas IL-10 enhances the production of TGF-β and also controls the ability of target cells to respond to TGF-β (29). We found that both TGF-β and IL-10 were secreted more by Tregs from HBV-HCC than non-HBV-HCC and thus would be more suppressive.

In chronic disease stage, PD1 pathway normally gets activated and is involved in promoting tolerance and preventing tissue damage (30). In our study, PD1 expression was less in Tregs in HBV-HCC than non-HBV-HCC patients. In non-HBVHCC, increased expression of PD1 on Tregs indicates that Tregs can be exhausted and their capability of suppression can be compromised.

In this study, HBV-HCC patients showed an increased expression of Notch 1 on Tregs from HBV-HCC compared to those from non-HBVHCC patients. Notch signaling helps in development and functioning of Tregs (10). *In vitro* studies have shown that blockade of Notch1 signaling inhibits Treg suppressor function (12).

In conclusion, Tregs in HBV-HCC patients are not only high in number but are also functionally more suppressive and positively correlate with AFP, and their mechanism of suppression through TGF-β. AFP value more than 100 thereby conferring an increased anti-tumor suppressive response as compared to non-HBV-HCC patients. Further, therapeutic interventions can consider Tregs: AFP correlation to characterize the HBV-HCC patients.

## Conflict of Interest Statement

The authors declare that the research was conducted in the absence of any commercial or financial relationships that could be construed as a potential conflict of interest.

## Supplementary Material

The Supplementary Material for this article can be found online at http://www.frontiersin.org/Journal/10.3389/fimmu.2015.00049/abstract

## Abbreviations

HBV, hepatitis B virus; HCC, hepatocellular carcinoma; TGF-β, transforming growth factor-β; Tregs, T regulatory cells.
