# Supplementary material for: CD4+CD25+CD127low Regulatory T Cells Play Predominant Anti-Tumor Suppressive Role in Hepatitis B Virus-Associated Hepatocellular Carcinoma
Source: Front Immunol. 2015 Feb 25;6:49. doi: 10.3389/fimmu.2015.00049 (PMC4341117; doi:10.3389/fimmu.2015.00049)
Supplement: Supplementary file 1 [file table_1.doc]

| **Supplementary Table 1. Correlation Coefficient in HBV HCC patients with HIGH AFP ( < 1000 )** | | | | | | | | | | |
| --- | --- | --- | --- | --- | --- | --- | --- | --- | --- | --- |
|  | | | **AFP** | **CD4+ CD25hi** | | **Foxp3 in CD4+ CD25hi CD127-** | **CD4+ CD25hi CD127-** | **PD1 in CD4+ CD25hi** | **IL-10 in CD4+ CD25hi** | **TGF-β in CD4+ CD25hi** |
| Spearman's rho | **AFP** | r | 1.000 | -.401 | | **.857^*^** | .000 | -.667 | .286 | -.738 |
|  |  | P value | . | .373 | | **.014** | 1.000 | .102 | .535 | .262 |
|  |  |  |  |  |  |  |  |  |  |  |
|  | **CD4+ CD25hi** | r | -.401 | | 1.000 | -.401 | .408 | -.231 | -.223 | . |
|  |  | P value | .373 | | . | .373 | .363 | .618 | .631 | . |
|  |  |  |  |  |  |  |  |  |  |  |
|  | **Foxp3 in CD4+ CD25hi CD127-** | r | **.857^*^** | | -.401 | 1.000 | .327 | -.778^*^ | -.143 | .105 |
|  |  | P value | **.014** | | .373 | . | .474 | .039 | .760 | .895 |
|  |  |  |  |  |  |  |  |  |  |  |
|  | **CD4+ CD25hi CD127-** | r | .000 | .408 | | .327 | 1.000 | -.472 | -.618 | .056 |
|  |  | P value | 1.000 | .363 | | .474 | . | .285 | .139 | .944 |
|  |  |  |  |  |  |  |  |  |  |  |
|  | **PD1 in CD4+ CD25hi** | r | -.667 | -.231 | | **-.778^*^** | -.472 | 1.000 | .185 | .056 |
|  |  | P value | .102 | .618 | | **.039** | .285 | . | .691 | .944 |
|  |  |  |  |  |  |  |  |  |  |  |
|  | **IL-10 in CD4+ CD25hi** | r | .286 | -.223 | | -.143 | -.618 | .185 | 1.000 | -.949 |
|  |  | P value | .535 | .631 | | .760 | .139 | .691 | . | .051 |
|  |  |  |  |  |  |  |  |  |  |  |
|  | **TGF-β in CD4+ CD25hi** | r | -.738 | . | | .105 | .056 | .056 | **-.949** | 1.000 |
|  |  | P value | .262 | . | | .895 | .944 | .944 | **.051** | . |
|  |  |  |  |  |  |  |  |  |  |  |
| *. Correlation is significant at the 0.05 level (2-tailed). | | | | | | | | | | |
