# Supplementary material for: CD4+CD25+CD127low Regulatory T Cells Play Predominant Anti-Tumor Suppressive Role in Hepatitis B Virus-Associated Hepatocellular Carcinoma
Source: Front Immunol. 2015 Feb 25;6:49. doi: 10.3389/fimmu.2015.00049 (PMC4341117; doi:10.3389/fimmu.2015.00049)
Supplement: Supplementary file 2 [file table_2.doc]

| **Supplementary Table 2. Correlation Coefficient in HBV HCC patients with low AFP ( >1000 )** | | | | | | | | | | |
| --- | --- | --- | --- | --- | --- | --- | --- | --- | --- | --- |
|  | | | **AFP** | **CD4+ CD25hi** | **Foxp3 in CD4+ CD25hi CD127-** | **CD4+ CD25hi CD127-** | **PD1 in CD4+ CD25hi** | **IL-10 in CD4+ CD25hi** | **TGF-β in CD4+ CD25hi** |  |
| Spearman's rho | **AFP** | r | 1.000 | .092 | .182 | .444 | .379 | .262 | -.462 |  |
|  |  | P value | . | .800 | .614 | .199 | .280 | .464 | .434 |  |
|  |  |  |  |  |  |  |  |  |  |  |
|  | **CD4+ CD25hi** | r | .092 | 1.000 | .148 | -.059 | .371 | .281 | .216 |  |
|  |  | P value | .800 | . | .682 | .871 | .291 | .431 | .727 |  |
|  |  |  |  |  |  |  |  |  |  |  |
|  | **Foxp3 in CD4+ CD25hi CD127-** | r | .182 | .148 | 1.000 | .555 | .555 | **.914^**^** | .079 |  |
|  |  | P value | .614 | .682 | . | .096 | .096 | **.000** | .900 |  |
|  |  |  |  |  |  |  |  |  |  |  |
|  | **CD4+ CD25hi CD127-** | r | .444 | -.059 | .555 | 1.000 | .605 | .465 | -.308 |  |
|  |  | P value | .199 | .871 | .096 | . | .064 | .176 | .614 |  |
|  |  |  |  |  |  |  |  |  |  |  |
|  | **PD1 in CD4+ CD25hi** | r | .379 | .371 | .555 | .605 | 1.000 | .406 | -.026 |  |
|  |  | P value | .280 | .291 | .096 | .064 | . | .244 | .966 |  |
|  |  |  |  |  |  |  |  |  |  |  |
|  | **IL-10 in CD4+ CD25hi** | r | .262 | .281 | .914^**^ | .465 | .406 | 1.000 | -.359 |  |
|  |  | P value | .464 | .431 | .000 | .176 | .244 | . | .553 |  |
|  |  |  |  |  |  |  |  |  |  |  |
|  | **TGF-β in CD4+ CD25hi** | r | -.462 | .216 | .079 | -.308 | -.026 | -.359 | 1.000 |  |
|  |  | P value | .434 | .727 | .900 | .614 | .966 | .553 | . |  |
|  |  |  |  |  |  |  |  |  |  |  |
| **. Correlation is significant at the 0.01 level (2-tailed). | | | | | | | | | |  |
