# Supplementary material for: CD4+CD25+CD127low Regulatory T Cells Play Predominant Anti-Tumor Suppressive Role in Hepatitis B Virus-Associated Hepatocellular Carcinoma
Source: Front Immunol. 2015 Feb 25;6:49. doi: 10.3389/fimmu.2015.00049 (PMC4341117; doi:10.3389/fimmu.2015.00049)
Supplement: Supplementary file 3 [file table_3.doc]

| **Supplementary Table 3. Correlation Coefficient in NON- HBV HCC patients with low AFP ( >1000 )** | | | | | | | | | |
| --- | --- | --- | --- | --- | --- | --- | --- | --- | --- |
|  | | | **AFP** | **CD4+ CD25hi** | **Foxp3 in CD4+ CD25hi CD127-** | **CD4+ CD25hi CD127-** | **PD1 in CD4+ CD25hi** | **IL-10 in CD4+ CD25hi** | **TGF-β in CD4+ CD25hi** |
| Spearman's rho | **AFP** | r | 1.000 | -.668^**^ | .060 | .124 | -.225 | -.379 | .329 |
|  |  | P Value | . | .005 | .826 | .647 | .403 | .148 | .353 |
|  |  |  |  |  |  |  |  |  |  |
|  | **CD4+ CD25hi** | r | -.668^**^ | 1.000 | -.053 | -.154 | .044 | .353 | -.344 |
|  |  | P value | .005 | . | .823 | .516 | .853 | .165 | .330 |
|  |  |  |  |  |  |  |  |  |  |
|  | **Foxp3 in CD4+ CD25hi CD127-** | Correlation Coefficient | .060 | -.053 | 1.000 | .282 | .319 | -.251 | .220 |
|  |  | P value | .826 | .823 | . | .228 | .171 | .330 | .542 |
|  |  |  |  |  |  |  |  |  |  |
|  | **CD4+ CD25hi CD127-** | Correlation Coefficient | .124 | -.154 | .282 | 1.000 | .341 | .187 | -.488 |
|  |  | P value | .647 | .516 | .228 | . | .141 | .471 | .153 |
|  |  |  |  |  |  |  |  |  |  |
|  | **PD1 in CD4+ CD25hi** | Correlation Coefficient | -.225 | .044 | .319 | .341 | 1.000 | .469 | -.314 |
|  |  | P value | .403 | .853 | .171 | .141 | . | .058 | .377 |
|  |  |  |  |  |  |  |  |  |  |
|  | **IL-10 in CD4+ CD25hi** | Correlation Coefficient | -.379 | .353 | -.251 | .187 | .469 | 1.000 | -.449 |
|  |  | P value | .148 | .165 | .330 | .471 | .058 | . | .193 |
|  |  |  |  |  |  |  |  |  |  |
|  | **TGF-β in CD4+ CD25hi** | Correlation Coefficient | .329 | -.344 | .220 | -.488 | -.314 | -.449 | 1.000 |
|  |  | P value | .353 | .330 | .542 | .153 | .377 | .193 | . |
|  |  |  |  |  |  |  |  |  |  |
| **. Correlation is significant at the 0.01 level (2-tailed). | | | | | | | | | |
