# Supplementary figures and images for: CD4+CD25+CD127low Regulatory T Cells Play Predominant Anti-Tumor Suppressive Role in Hepatitis B Virus-Associated Hepatocellular Carcinoma
Source: Front Immunol. 2015 Feb 25;6:49. doi: 10.3389/fimmu.2015.00049 (PMC4341117; doi:10.3389/fimmu.2015.00049)

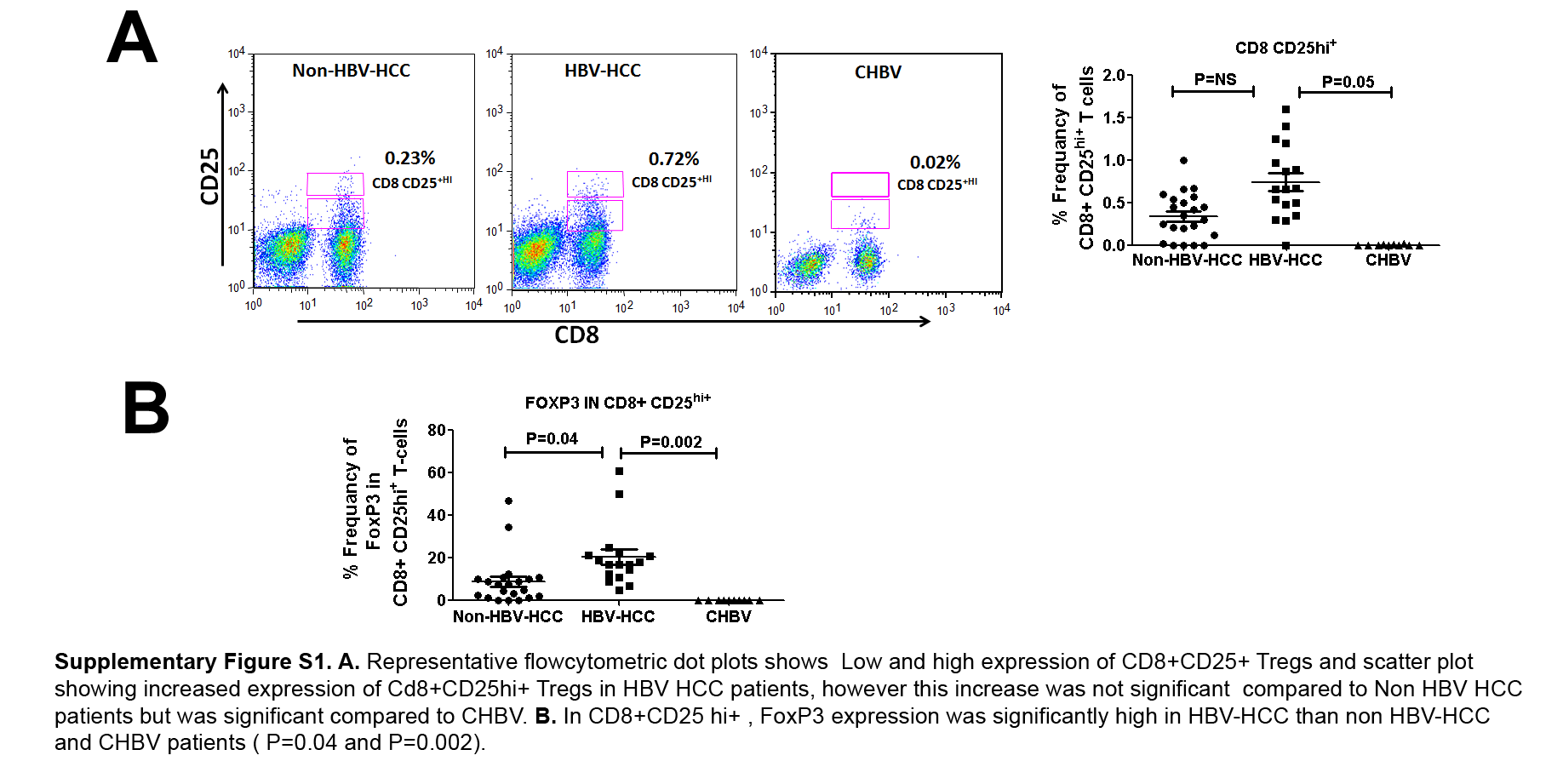

Supplement: Supplementary file 4 [file image_1.tif]

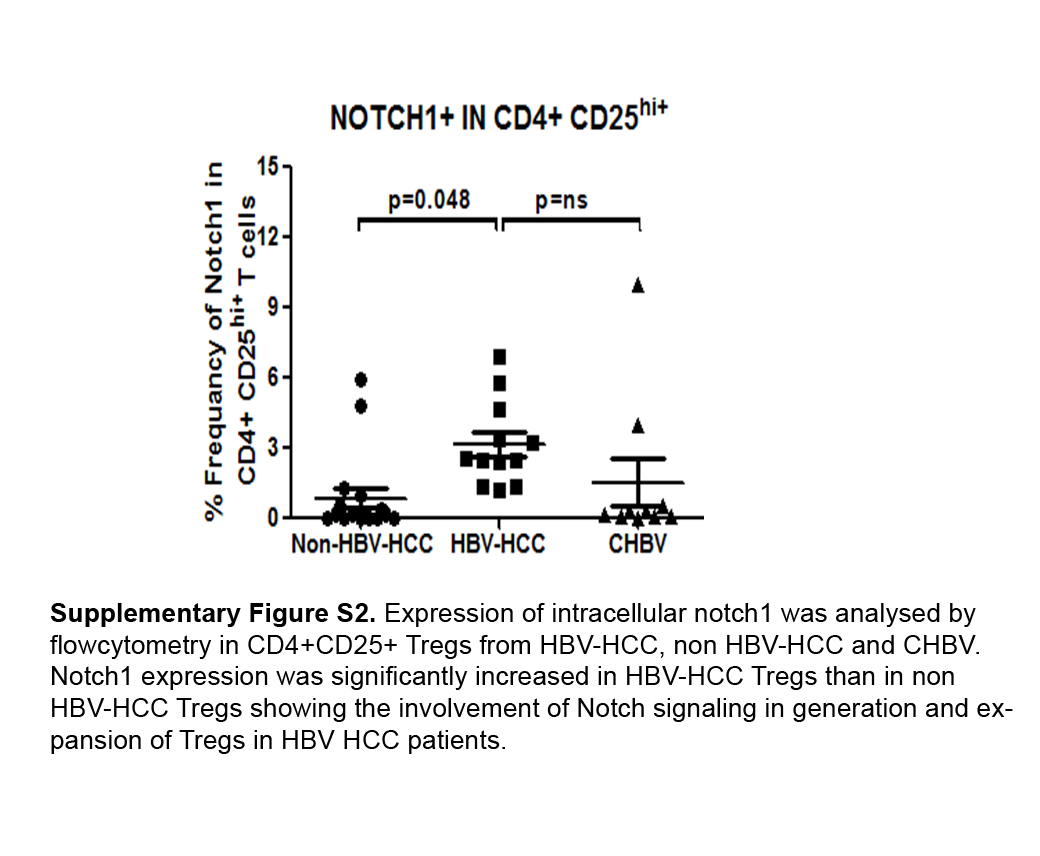

Supplement: Supplementary file 5 [file image_2.tif]
